# Risk Factors of Microscopically Tumor-Free Surgical Margins for Recurrence and Survival of Oral Squamous Cell Carcinoma Patients

**DOI:** 10.3389/fonc.2022.930988

**Published:** 2022-07-07

**Authors:** Meiling Pei, Dawool Han, Ki-Yeol Kim, Dong Wook Kim, Woong Nam, Hyung Jun Kim, Eunae Sandra Cho, Hyun Sil Kim, In-Ho Cha, Xianglan Zhang

**Affiliations:** ^1^ Department of Dermatology, Yonsei University College of Dentistry, Seoul, South Korea; ^2^ Department of Oral Pathology, Yonsei University College of Dentistry, Seoul, South Korea; ^3^ BK21 FOUR Project, Yonsei University College of Dentistry, Seoul, South Korea; ^4^ Department of Dental Education, Yonsei University College of Dentistry, Seoul, South Korea; ^5^ Department of Oral and Maxillofacial Surgery, Yonsei University College of Dentistry, Seoul, South Korea; ^6^ Department of Pathology, Yanbian University Hospital, Yanji, China; ^7^ Oral Cancer Research Institute, Yonsei University College of Dentistry, Seoul, South Korea

**Keywords:** OSCC, surgical margin, risk assessment, molecular markers, axin2, snail, recurrence, prognosis

## Abstract

**Objectives:**

The concept of adequate surgical margins remains controversial in oral squamous cell carcinoma (OSCC) surgery. This study aimed to identify surgical margin-related indicators that might impact recurrence and survival of OSCC patients.

**Materials and Methods:**

Histopathological examination was performed using hematoxylin-eosin-stained surgical margin tissue sections in 235 OSCC patients. Axin2 and Snail expression at the surgical margin was detected by immunohistochemistry. The impact of the Axin2-Snail cascade on tumorigenesis of the immortalized human oral keratinocyte (IHOK) line was investigated *in vivo*.

**Results:**

The width and dysplasia of surgical margins were not significantly associated with the outcome of OSCC patients. In a multivariate analysis using variable clinicopathologic factors and with Axin2 and Snail expression as cofactors, higher age (hazard ratio [HR]:1.050; *P*=0.047), Axin2 (HR:6.883; *P*=0.014), and Snail abundance (HR:5.663; *P*=0.009) had independent impacts on worsened overall survival. Similarly, lesion site in retromolar trigone (HR:4.077; *P*=0.010), upper (HR:4.332; *P*=0.005) and lower gingiva (HR:3.545; *P*=0.012), presence of extranodal extension (HR:9.967; *P*<0.001), perineural invasion (HR:3.627; *P*=0.024), and Snail abundance (HR:3.587; *P*<0.001) had independent impacts on worsened recurrence-free survival. Furthermore, Axin2 knockdown induced decreased Snail expression and attenuated tumorigenesis in the IHOK line.

**Conclusion:**

Histopathological examination of surgical margins may not be reliable to predict OSCC patient outcome. Molecular analysis may provide a more accurate risk assessment of surgical margins in OSCC. In particular, Axin2 and Snail are potential predictive biomarkers for the risk assessment of surgical margins in OSCC.

## Introduction

As the most common histologic type of oral cancer, oral squamous cell carcinoma (OSCC) accounts for approximately 90% of oral cavity malignancies (1). More than 200000 new cases of OSCC are reported annually worldwide (2), and the five-year survival rate of patients is only ~50% (3). Despite good diagnostic and therapeutic strategies, local recurrence or distant metastasis occurs in 40–60% of OSCC patients (4, 5).

Surgical resection is the most accepted choice for OSCC therapy, and the characteristics of surgical margins in OSCC are considered a powerful predictive factor for both recurrence and patient survival (6–10). During OSCC surgery, surgeons should aim not only to secure sufficient resection margins, but also to maintain an acceptable physical appearance and the functions of adjacent organs of the oral cavity. Therefore, most OSCC patients have insufficient margins after surgical resection.

In OSCC surgery, the concept of marginal status (adequate or inadequate) remains controversial. Traditionally, the adequacy of margins was determined based on the width of the resection margin, defined as the distance from the histologically confirmed tumor edge to the inked margin of the specimen. The Royal College of Pathologists (PCRath)-issued guidelines are the most widely accepted criteria for histological OSCC resection margin status (11). In the PCRath scoring system, resection margins are divided into three categories based on the width of the resection margins: clear (width > 5 mm), close (width = 1–5 mm), and positive (width < 1 mm) margins. Generally, a clear margin is denoted as adequate, and both close and positive margins are considered inadequate. However, various factors, such as tissue shrinkage and mucosal elasticity, can influence the width of the resection margins postoperatively (12, 13). In particular, the degree of shrinkage varies according to many factors, including tissue component, lesion site, stage of tumors, status of keratinization, inflammation, and pathological processing (13). Thus, there is a considerable discrepancy in the width between the clinical and pathological resection margins. Therefore, it is difficult to represent the status of resection margins in patients with OSCC based only on the width of the pathological margins.

Research on the molecular characteristics of surgical margins is ongoing. In 1953, Slaughter et al. observed that histologically altered cells were present in tissues adjacent to cancers, and termed this phenomenon ‘field cancerization’ (14). Recently, researchers have found that ‘field cancerization’ is induced by various genetic alterations, such as gene mutation and amplification, as well as aberrant methylations; some of the histologically normal cells adjacent to cancer tissues also show genetic alterations (15–17). The aberrant expression of p53 and p16 in histologically normal surgical margins of OSCC has been highlighted by several investigators (15, 18). However, predictive biomarkers currently fall short of estimating the status of surgical margins of OSCC.

The crucial role of epithelial-to-mesenchymal transition (EMT), a well-known biological process, in both the invasion and metastasis of various cancers, including OSCC, has been well established (19). Moreover, the possible implications of EMT genes, such as axis inhibition protein 2 (Axin2) and Snail, in early-stage carcinogenesis was also highlighted in a previous study (20). Significantly increased Snail expression has been observed in early-stage endometrial carcinoma before invasion. In addition, overexpression of both Axin2 and Snail can be found in some precancerous lesions, including colorectal adenoma and oral leukoplakia (21–23). In our previous study, we also found that overexpression of Axin2 and Snail is a potential risk factor for the malignant conversion of oral leukoplakia (23). In this study, we determined the predictive value of Axin2 and Snail expression in the risk assessment of the surgical margin of OSCC and further evaluated the impact of the Axin2-Snail cascade on the tumorigenic activity of a spontaneously immortalized human oral keratinocyte (IHOK) line (24) *in vivo*. Histopathological risk assessment was also performed in our OSCC cohort based on the evaluation of the width and presence of epithelial dysplasia (ED) in the surgical margins.

## Materials and Methods

### Patients in the OSCC Cohort

**Figure 1:**
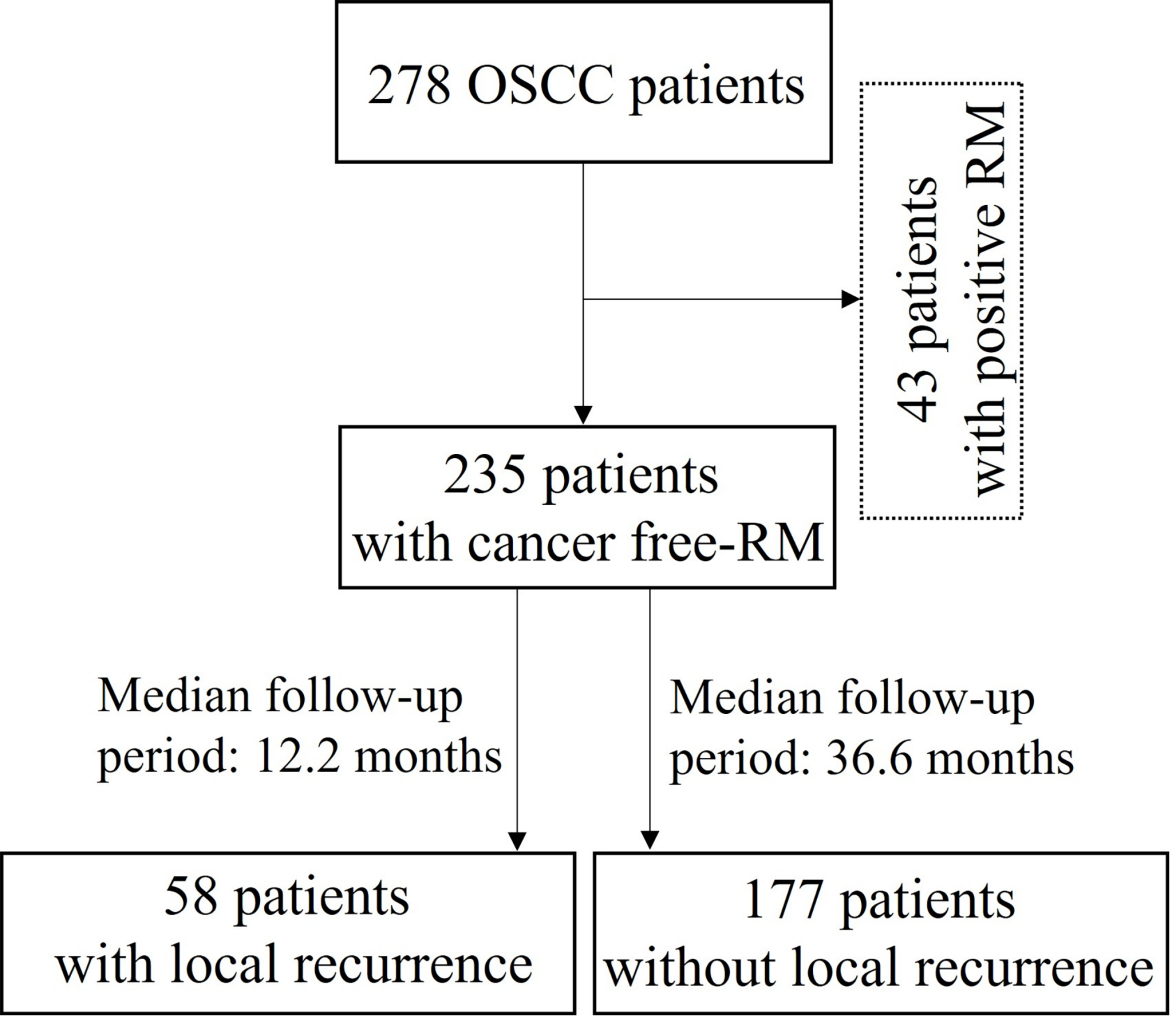
Flow diagram for the selection and outcome of patients with oral squamous cell carcinoma (OSCC: Oral squamous cell carcinoma; RM: Resection margin).

A total of 278 patients with OSCC who underwent surgery at the Department of Oral and Maxillofacial Surgery, Dental Hospital, Yonsei University Medical Center, between 2009 and 2018 were retrospectively reviewed; 43 patients with tumor extension to the surface of resection margin were excluded from this study. Tissue samples of the surgical resection margin were obtained from 235 OSCC patients from the Department of Oral Pathology: 58 (24.7%) patients with recurrence (median follow-up period: 12.2 months) and 177 (75.3%) patients without recurrence during follow-up (median follow-up: 36.6 months) (**Figure 1**). Patient characteristics are shown in **Table 1**. This study was approved by the Institutional Review Board of the Yonsei University College of Dentistry (2-2021-0098).

**Table 1:** Clinicopathologic characteristics of 235 OSCC patients.

Variables	No. patients (%)
Total cases	235
*Age*	
Median age (range)	60 (23–91)
<60	118 (50.2)
≥60	117 (49.8)
*Gender*	
Male	131 (55.7)
Female	104 (44.3)
*Lesion site*	
Tongue	78 (33.2)
RMT	13 (5.5)
Upper gingiva	41 (17.4)
Lower gingiva	55 (23.4)
FOM	13 (5.5)
Buccal cheek	31 (13.2)
Lip	4 (1.7)
*T stage*	
T1	72 (30.6)
T2	76 (32.3)
T3	20 (8.5)
T4	67 (28.5)
*LN status*	
N0	176 (74.9)
N1	17 (7.2)
N2	27 (11.5)
N3	15 (6.4)
*Extranodal extension*	
Absent	213 (90.6)
Present	22 (9.4)
*Histological grade*	
WD	36 (15.3)
MD	162 (68.9)
PD	37 (15.7)
*Peri-neural invasion*	
Absent	212 (90.2)
Present	23 (9.8)
*Vascular invasion*	
Absent	210 (89.4)
Present	23 (9.8)
*Dysplasia in RM*	
Absent	172 (73.2)
Present	63 (26.8)
*Width of RM*	
<1mm	36 (15.3)
≥1mm to <3mm	108 (46.0)
≥3mm to <5mm	76 (32.3)
≥5mm	15 (6.4)

FOM, Floor of mouth; RMT, Retromolar trigone; LN, lymph node; WD, Well differentiated; MD, Moderately differentiated; PD, Poorly differentiated; RM, Resection margin.

### Immunohistochemical Staining

Immunohistochemistry was performed on 4-μm sections of paraffin-embedded tissue specimens of surgical margins obtained from 235 patients with OSCC. Axin2 (Rabbit polyclonal IgG, working dilution 1:500; ab32197, Abcam, Cambridge, UK) and Snail antibodies (Rabbit polyclonal IgG, working dilution 1:1000) were used as primary antibodies. The Snail antibody was prepared as previously described (25). Endogenous peroxidase activity was inhibited using a mixture of H_2_O_2_ and methanol (1:40), and antigen retrieval was performed by the pressure-cooking method using citrate buffer (pH 6.0, Sigma-Aldrich, Darmstadt, Germany) for the deparaffinized tissue sections. The Real Envision HRP Rabbit/Mouse detection system (Dako, Glostrup, Denmark) was used as a secondary antibody, and immunoreactivity of the tissue sections against Axin2 and Snail was visualized using 3,3′-diaminobenzidine.

The histoscore for the immunoreactivity of both Axin2 and Snail in the surgical margins of OSCC was calculated according to the staining intensity and percentage of positive cells using the weighted histoscore method (26). Patients were subdivided into two groups according to the histoscore: low (histoscore 0–100) and high (histoscore 101–300) expression groups.

### Cell Culture and Establishment of Axin2 Knockdown IHOK Line

IHOKs were cultured in a medium composed of Dulbecco’s modified Eagle’s medium (DMEM; Gibco BRL, USA) and Ham’s nutrient mixture F-12 (Ham’s F12; Gibco BRL, USA) at a ratio of 3:1. The medium was supplemented with 10% Tet-approved FBS (HyClone Laboratories, Inc., Logan, UT, USA), 1% penicillin/streptomycin (Sigma-Aldrich), 0.01 µg/mL cholera toxin (Sigma-Aldrich), 0.04 µg/mL hydrocortisone (Sigma-Aldrich), 0.5 µg/mL insulin, 0.5 µg/mL apo-transferrin(Sigma-Aldrich), and 0.2 µg/mL 3′-5-triodo-1-thyroine (Sigma-Aldrich). The pLKO-Tet-shAxin2 vector was constructed using the pLKO-Tet-On vector (Addgene, Cambridge, MA, USA) and Axin2 oligo (5’-ACCACCACTACATCCACCA-3’). The Axin2 knockdown IHOK line was constructed by transfection of the pLKO-Tet-shAxin2 vector as well as treatment with doxycycline (5 μg/mL, Sigma-Aldrich).

### Western Blot Analysis

Western blot analysis was performed in both the pLKO-Tet-ShAxin2-transfected stable IHOK line with or without doxycycline treatment and tumor nodules obtained from the animal study. Total protein extraction was performed with lysis buffer (RIPA buffer; Cell Signaling Technology, Inc., MA, USA), and a 10% sodium dodecyl sulfate-polyacrylamide gel electrophoresis system was used for protein resolution. After the proteins were transferred to polyvinylidene difluoride membranes (EMD Millipore, MA, USA), blocking was performed using 5% non-fat milk. Primary antibodies against Axin2 (Abcam), Snail, and glyceraldehyde-3-phosphate dehydrogenase (GAPDH; Cell Signaling Technology) were used for western blot analysis, and the related signal was developed using an enhanced chemiluminescence detection system (Pierce Biotechnology, IL, USA).

### Tumor Growth in Mice

Ten female 6-week-old BALB/c-nu/nu mice (BALB/c Slc-nu/nu; Japan SLC, Inc., Hamamatsu, Japan) were randomized into two groups (n=5 per group). A total of 5 × 10^5^ pLKO-Tet-ShAxin2-transfected IHOK cells were subcutaneously injected into the two groups of mice. For the experimental group, cells were pretreated with 5 μg/mL doxycycline for 48 h before cell injection. After cell injection, the experimental group was administered doxycycline at 10 mg/kg body weight by intraperitoneal injection for five consecutive days per week for 28 days. The control group was administered phosphate-buffered saline (pH 7.4) according to the same schedule as that of the experimental group. The tumor volume was determined every 4 days and calculated by measuring the length and width of the tumor nodules (27). At the end of the study period, all mice were sacrificed, and the tumor nodules were removed. All procedures for the animal study were performed according to protocols approved by the Animal Care and Use Committee of the College of Medicine of Yonsei University. The animal research program used in the present study was accredited by the Association for Assessment and Accreditation of Laboratory Animal Care International.

### Statistical Analysis

The difference in the volume of tumor nodules between groups was assessed using the Mann–Whitney *U* test. The Chi-square test, Kaplan–Meier analysis, and Cox regression analysis were performed to estimate the clinicopathological relevance of surgical margin characteristics in the OSCC cohort. Results were considered statistically significant at *P* < 0.05. The SPSS 25 statistical package (SPSS, Inc., Chicago, IL, USA) was used for statistical analysis.

## Results

### Histopathologic Assessment for Resection Margins in the OSCC Cohort

**Figure 2:**
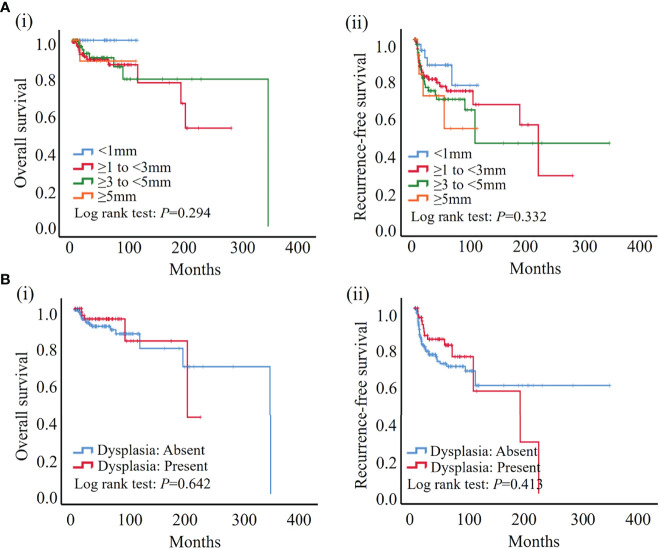
According to the results of the Kaplan–Meier analysis, neither width nor dysplasia was significantly associated with patient outcome. **(A)** No significant association was found between width of surgical margin and both overall **(i)** and recurrence-free survival **(ii)** in our cohort. **(B)** No significant association was found between dysplasia of surgical margin and both overall **(i)** and recurrence-free survival **(ii)** in our cohort.

Histopathologic assessment was performed independently by two pathologists by evaluating the width and presence of epithelial dysplasia in the hematoxylin-eosin-stained surgical margin of OSCC patients. In this study, margins of 1, 3, and 5 mm were set as the commonly used margin cut-offs to specify adequate safety margins based on previous literature (9, 28–30). In our cohort, 36 (15.3%), 108 (46.0%), 76 (32.3%), and 15 (6.4%) patients showed <1 mm, ≥1 mm to <3 mm, ≥3 mm to <5 mm, and ≥5 mm resection margin widths, respectively. Dysplastic surgical margins were found in 63 (26.8%) patients, and which 6 (9.5%) patients showed severe dysplasia in our cohort. According to the Kaplan–Meier analysis, neither the width of the surgical margin nor margins with dysplasia was significantly associated with both recurrence-free and overall survival of the patients in our cohort (**Figure 2**).

### Axin2 and Snail Are Potential Novel Biomarker(s) in the Risk Assessment of Surgical Margins in OSCC

**Figure 3:**
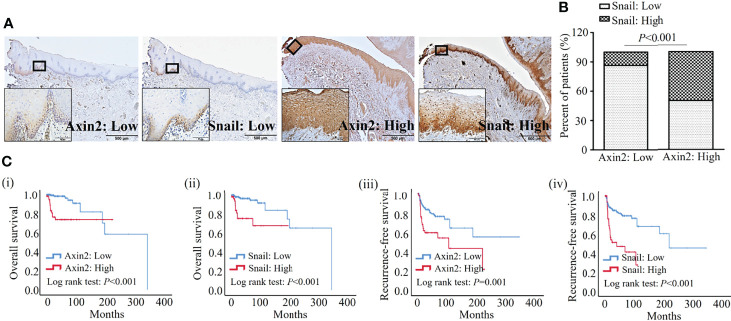
Clinicopathologic significance of Axin2 and Snail expression in surgical margins from the OSCC cohort. **(A)** Representative expression patterns for low or high levels of Axin2 and Snail in surgical margins of OSCC; **(B)** Axin2 and Snail expression showed a significant correlation in surgical margins of OSCC; **(C)** Axin2 and Snail expression in surgical margins showed significant associations with overall and recurrence-free survival in OSCC patients.

Cytoplasmic Axin2 expression was found in epithelial cells of resection margins in OSCC, and tissue immunoreactivity against Axin2 was high in 60 (Axin2-high, 25.5%) and low in 175 (Axin2-low, 74.5%) surgical margin tissue samples. In contrast, Snail expression was found in both the nucleus and cytoplasm of epithelial cells in surgical margins of OSCC, and the immunoreactivity against Snail was high in 54 (Snail-high, 23.0%) and low in 181 (Snail-low, 77.0%) surgical margin tissue samples. Representative expression patterns for low or high immunoreactivity for both Axin2 and Snail in the surgical margins are shown in **Figure 3A**. Interestingly, Snail-high expression was detected more often in resection margins with Axin2-high expression (30, 50.0%) than in resection margins with Axin2-low expression (24, 13.7%) (*P*<0.001) (**Figure 3B**). Consistent with previous studies, Axin2-mediated Snail stabilization may also be present in the resection margins of OSCC (25).

In our cohort, no significant association was found between the width or presence of dysplasia in resection margins and both Axin2 and Snail expression (**Table 2**). Meanwhile, according to Kaplan-Meir analysis, both Axin2 and Snail abundance was significantly associated with both overall (both *P*<0.001) and recurrence-free survival (*P*=0.001 and *P*<0.001) in our cohort (**Figure 3C**). In a multivariate analysis using variable clinicopathologic factors and Axin2 and Snail as cofactors, higher age (Hazard ratio, HR: 1.050; 95% confidence interval, CI: 1.001–1.102; *P*=0.047), Axin2 (HR: 6.883; 95% CI: 1.467–32.284; *P*=0.014), and Snail abundance (HR: 5.663; 95% CI: 1.555–20.619; *P*=0.009) had independent impacts on worsened overall survival of the patients, and lesion site in retromolar trigone (HR: 4.077; 95% CI: 1.409–11.791; *P*=0.010), upper– (HR: 4.332; 95% CI: 1.549–12.114; *P*=0.005) and lower gingiva (HR: 3.545; 95% CI: 1.315–9.555; *P*=0.012), presence of extranodal extension (HR: 9.967; 95% CI: 2.841–34.964; *P*<0.001), perineural invasion (HR: 3.627; 95% CI: 1.189–11.059; *P*=0.024), and Snail abundance (HR: 3.587; 95% CI: 1.839–6.998; *P*<0.001) in resection margins had independent impacts on worsened recurrence-free survival of OSCC patients (**Table 3**). However, there are no significant association between the width or presence of dysplasia in resection margins and both Axin2 and Snail expression (**Table 2**).

**Table 2:** Association between status of surgical margin and both Axin2 and Snail expression in 235 OSCC surgical margins.

Variables	Total, n (%)	Axin2		*P*	Snail		*P*
		Low	High		Low	High	
*Dysplasia in RM*							
Absent	172 (73.2)	128 (74.4)	44 (25.6)	0.977	136 (79.1)	36 (20.9)	0.217
Present	63 (26.8)	47 (74.6)	16 (25.4)		45 (71.4)	18 (28.6)	
*Width of RM*							
<1mm	36 (15.3)	29 (80.6)	7 (19.4)	0.491	29 (80.6)	7 (19.4)	0.901
≥1mm to <3mm	108 (46.0)	81 (75.0)	27 (25.0)		84 (77.8)	24 (22.2)	
≥3mm to <5mm	76 (32.3)	56 (73.7)	20 (26.3)		57 (75.0)	19 (25.0)	
≥5mm	15 (6.4)	9 (60.0)	6 (40.0)		11 (73.3)	4 (26.7)	

RM, Resection margin.

**Table 3:** Multivariable Cox-regression analysis for risk factors of overall and recurrence-free survival of 235 OSCC patients.

Variables	Overall survival		Recurrence–free survival	
	Hazard ratio (95% CI)	*P*	Hazard ratio (95% CI)	*P*
*Age*	1.050 (1.001–1.102)	0.047	1.015 (0.991–1.039)	0.232
*Gender*				
Male	1		1	
Female	0.502 (0.153–1.650)	0.257	0.928 (0.500–1.722)	0.812
*Lesion site*				
Tongue	1		1	
RMT	5.844 (0.762–44.801)	0.089	4.077 (1.409–11.791)	0.01
Upper gingiva	7.936 (0.872–72.209)	0.066	4.332 (1.549–12.114)	0.005
Lower gingiva	1.640 (0.163–16.524)	0.675	3.545 (1.315–9.555)	0.012
FOM	4.478 (0.309–64.847)	0.272	0.613 (0.074–5.104)	0.651
Buccal cheek	3.776 (0.561–25.416)	0.172	1.526 (0.509–4.574)	0.45
Lip	0.000 (0.000–1.021)	0.99	5.545 (0.871–35.306)	0.07
*T stage*				
T1	1		1	
T2	0.445 (0.084–2.351)	0.34	0.800 (0.343–1.869)	0.607
T3	0.339 (0.030–3.815)	0.381	0.934 (0.284–3.071)	0.911
T4	1.079 (0.174–6.682)	0.935	0.616 (0.226–1.682)	0.345
*LN status*				
N0	1		1	
N1	1.734 (0.273–11.001)	0.559	1.314 (0.495–3.486)	0.584
N2	0.837 (0.136–5.139)	0.848	0.360 (0.111–1.175)	0.091
N3	2.573 (0.254–26.024)	0.424	0.210 (0.043–1.022)	0.053
Extranodal extension				
Absent	1		1	
Present	0.848 (0.103–6.967)	0.878	9.967 (2.841–34.964)	<0.001
*Histological grade*				
WD	1		1	
MD	1.685 (0.288–9.869)	0.563	1.246 (0.523–2.968)	0.62
PD	6.384 (0.886–45.996)	0.066	1.042 (0.348–3.119)	0.942
*Peri–neural invasion*				
Absent	1		1	
Present	3.954 (0.563–27.781)	0.167	3.627 (1.189–11.059)	0.024
*Vascular invasion*				
Absent	1		1	
Present	1.538 (0.207–11.453)	0.674	1.303 (0.441–3.852)	0.633
*Dysplasia in RM*				
Absent	1		1	
Present	0.929 (0.242–3.565)	0.915	0.821 (0.404–1.670)	0.586
*Width of RM*				
<1mm	1		1	
≥1mm to <3mm	288476.736 (0.000–1.115E97)	0.907	2.309 (0.780–6.841)	0.131
≥3mm to <5mm	163863.292 (0.000–6.334E96)	0.911	2.235 (0.717–6.973)	0.166
≥5mm	131728.779 (0.000–5.176E96)	0.913	3.586 (0.697–18.454)	0.127
Axin2				
Low	1		1	
High	6.883 (1.467–32.284)	0.014	1.709 (0.866–3.369)	0.122
Snail				
Low	1		1	
High	5.663 (1.555–20.619)	0.009	3.587 (1.839–6.998)	<0.001

FOM, Floor of mouth; RMT, Retromolar trigone; WD, Well differentiated; MD, Moderately differentiated; PD, Poorly differentiated; RM, Resection margin.

### Axin2–Snail Cascade May Strongly Influence the Tumorigenesis of IHOK Line *in Vivo*

**Figure 4:**
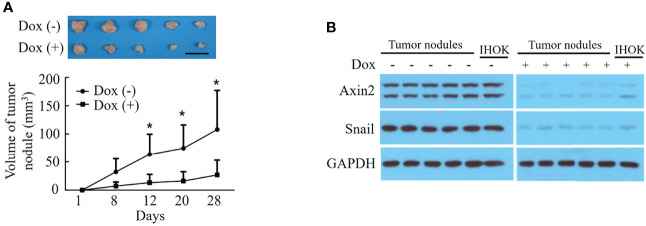
Knockdown of Axin2 attenuates tumorigenic activity in the IHOK line. **(A)** The volume of the tumor nodules was significantly lower in the doxycycline-treated group than in the control group (*P=0.032, Scale bar: 1cm). **(B)** Western blot analysis revealed decreased Axin2 and Snail expression both in the pLKO-Tet-shAxin2 vector-transfected IHOK line and tumor nodules from groups treated with doxycycline compared with that in controls.

The volume of the tumor nodules was significantly lower in the doxycycline-treated group than in the control group (*P*=0.032) (**Figure 4A**). Western blot analysis revealed decreased Axin2 and Snail expression both in the pLKO-Tet-shAxin2 vector-transfected IHOK line and tumor nodules from groups treated with doxycycline compared with that in controls (**Figure 4B**). The Axin2–Snail cascade may strongly influence tumorigenic activity in the IHOK line.

## Discussion

Risk assessment for surgical margins is necessary for the post-operative clinical management of patients with OSCC. In current clinical practice, width and dysplasia are routine histopathologic risk assessment points for surgical margins of OSCC.

Archiving additional histologically normal tissues at least 5 mm beyond the tumor tissue is generally considered the ‘gold standard’ in OSCC surgery. However, owing to the anatomical restrictions of the oral cavity, surgeons cannot achieve a sufficient width of the surgical margin in most patients with OSCC during surgery (30–32). In addition, the width of the margins is shortened by various side effects after surgery, including shrinkage and mucosal elasticity; therefore, pathologic surgical margins larger than 5 mm were found in only a minority of patients with OSCC, according to previous retrospective cohort studies, and in agreement with the present findings (32–34). Moreover, many studies have shown that 5-mm cut offs are not significant in patient outcome, and some investigators have explained that the width of certain safety margins may also be less than 5 mm. Therefore, it may be inappropriate to use 5-mm resection margins in surgical specimens as the cut-off distance for demarcation between adequate and inadequate margins.

Many previous studies have investigated the prognostic relevance of the width of surgical margins and have recommended varying margin cut-offs ranging from 1 to 5 mm. However, these margin cut-offs may not be universally applicable to all OSCC cohorts (33, 35). In our cohort, the width of the surgical margin did not show any significant association with the outcome of OSCC patients when the patients were subdivided into four groups based on 1-, 3-, and 5-mm cut-offs. The width of the surgical margin alone may lack evidence-based background to predict patient outcomes.

ED is considered a high-risk lesion for malignant transformation in the oral cavity. However, opinions regarding the risk of surgical margins involving ED in OSCC lack uniformity. Some investigators have observed that dysplastic surgical margins have a negative impact on patient outcomes, regardless of severity (36). However, other investigators have shown that the impact of mild or moderate dysplastic surgical margins is comparable to that of clear margins on patient outcome (37). In our cohort, we found that dysplastic resection margins were not significantly associated with overall and recurrence-free survival in patients with OSCC. According to the results of a survey by the American Head and Neck Society members, resection margins involving dysplasia were regarded as a ‘positive margin’ in 17%, ‘negative margin’ in 76%, and ‘variation in situation’ in 7% of responders (38). Our department policy is to consider severe dysplasia as the tumor itself and re-reset it until clear margins are obtained as much as possible, so only a limited number of severe dysplastic margins were included in our cohort. The difference in management practice between the organization and the subjectivity in diagnosis of dysplasia between observers would largely influence the predictive value of the dysplastic margin in the outcome of OSCC patients.

ED was traditionally divided into three-tier grades such as mild, moderate, and severe, based on the status of the dysplastic alterations such as architectural and cytological changes (39). However, the diagnosis of ED according to this system is very subjective and therefore, results in decreased reproducibility. Recently, World Health Organization suggested a new binary grading system of ED, which classified it into low-grade and high-grade ED, to overcome the limitations of its traditional grading system. The previous categories of ED namely, mild ED was included in low-grade ED, and moderate/severe ED and carcinoma *in situ* were included in high-grade ED (39, 40). Although the diagnostic reproducibility of ED and predictability for its malignant transformation are increased in the new binary grading system, there is still a need for investigating the molecular characteristics of ED to supplement the grading system (39, 41).

Resection margins of cancer tissues can harbor genetically altered cells during crosstalk with tumor cells or continued exposure to carcinogens. Genetic alterations in resection margins may be an important trigger for recurrence and poor prognosis in OSCC patients. However, it is difficult to detect by routine histopathological examination when genetically altered cells without predominant morphological change. Histopathological examination of surgical margins is not reliable for predicting the prognosis of OSCC, and molecular analysis is a suitable method to address this issue.

**Figure 5:**
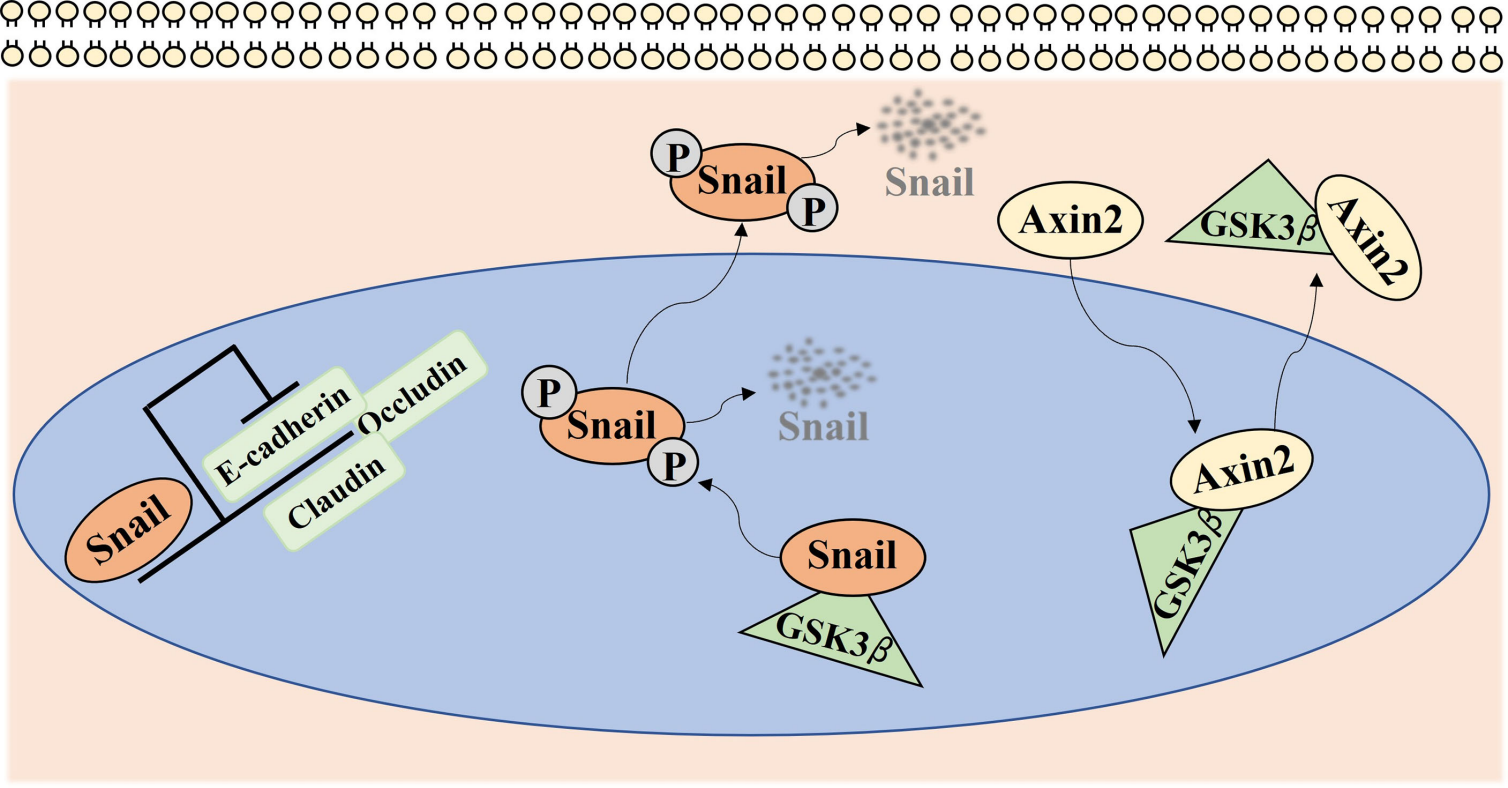
Axin2 expression implicates cancer progression *via* regulation of Snail mediated epithelial-mesenchymal transition. Snail, as a transcriptional repressor of many genes, such as E-cadherin, Claudin, and Occludin, mediates epithelial-mesenchymal transition in various types of cancers. GSK-3 can bind to and phosphorylate Snail, resulting in the subsequent degradation of Snail. As a GSK-3 scaffolding protein, Axin2 mediates the nucleocytoplasmic shuttle for GSK-3, resulting in the stabilization of nuclear Snail.

Axin2, as a target gene of the Wnt signaling pathway, was previously categorized as a tumor suppressor gene in a small fraction of colorectal cancers harboring APC gene mutations (42). However, recently, the oncogenic activities of Axin2 have been proposed in various types of cancers, such as breast, colorectal, and pancreatic cancers (43–45). Moreover, as a GSK-3 scaffolding protein, Axin2 is also known as an important participant in the stabilization of nuclear Snail because it mediates the nucleocytoplasmic shuttle for GSK-3, which can bind to and phosphorylate Snail, resulting in the subsequent degradation of Snail (**Figure 5**) (25). Snail, a transcriptional repressor of E-cadherin, is overexpressed in a variety of cancers and promotes the proliferation, invasion, and tumorigenesis of cancer cells (46, 47). Moreover, Axin2 and Snail expression were significantly associated with some of the classic poor prognostic indicators, such as lymph node metastasis, vascular invasion, and bone invasion in various types of cancers including OSCC (48–50). In our previous studies, we found that the Axin2–Snail axis is significantly related to malignant transformation of oral leukoplakia (23). Consistent with previous observations, we found that Axin2 knockdown induced decreased Snail expression as well as attenuated tumorigenic activity in IHOK cells. Moreover, Snail expression had a positive correlation with Axin2 expression in resection margins of OSCC, while both Axin2 and Snail were independent risk factors for overall survival of patients in our cohort when multiple clinicopathological factors, including age, sex, lesion site, histologic grade, T stage, N stage, extranodal extension, vascular invasion, perineural invasion, width, and dysplasia in resection margins were used as cofactors.

In summary, while some of the conventional clinical factors of OSCC, as well as Axin2 and Snail abundance in surgical margins, conferred a poor prognosis, both width and dysplasia, factors determined by histopathologic assessment for surgical margin, did not. Molecular analysis may provide a more accurate and objective assessment of surgical margins. Our results imply that the activation of EMT genes may be involved in the carcinogenesis cascade of surgical margins in OSCC. In particular, both Axin2 and Snail are possible biomarkers for resection margins for predicting outcomes in patients with OSCC.

## Data Availability Statement

The raw data supporting the conclusions of this article will be made available by the authors, without undue reservation.

## Ethics Statement

The studies involving human participants were reviewed and approved by Institutional Review Board of the Yonsei University College of Dentistry. The patients/participants provided their written informed consent to participate in this study. The animal study was reviewed and approved by Committee of the College of Medicine of Yonsei University.

## Author Contributions

Methodology, MP and DH; Software, MP and K-YK; Validation, DK, WN, and EC; Formal analysis, MP and K-YK; Investigation: MP, DH, and K-YK; Writing-original draft preparation: MP and DH; Writing-review and editing, HK, HK, I-HC, and XZ; Supervision, I-HC, XZ. All authors have read and agreed to the published version of the manuscript.

## Funding

This work was supported by a National Research Foundation of Korea (NRF) grant funded by the Korean government (MSIT) (No. 2020R1I1A1A01073437; No. 2021R1A2C1094413) and Yonsei University College of Dentistry Fund (No. 6-2019-0017).

## Conflict of Interest

The authors declare that the research was conducted in the absence of any commercial or financial relationships that could be construed as a potential conflict of interest.

## Publisher’s Note

All claims expressed in this article are solely those of the authors and do not necessarily represent those of their affiliated organizations, or those of the publisher, the editors and the reviewers. Any product that may be evaluated in this article, or claim that may be made by its manufacturer, is not guaranteed or endorsed by the publisher.

## Abbreviations

EMT, epithelial-to-mesenchymal transition; HR, hazard ratio; IHOK, immortalized human oral keratinocyte; OSCC, oral squamous cell carcinoma; RM, resection margin.
